# Genome-Wide Transcriptome Analyses of Silicon Metabolism in *Phaeodactylum tricornutum* Reveal the Multilevel Regulation of Silicic Acid Transporters

**DOI:** 10.1371/journal.pone.0007458

**Published:** 2009-10-14

**Authors:** Guillaume Sapriel, Michelle Quinet, Marc Heijde, Laurent Jourdren, Véronique Tanty, Guangzuo Luo, Stéphane Le Crom, Pascal Jean Lopez

**Affiliations:** 1 Biomineralization and Morphogenesis Group, CNRS UMR-8186, Ecole Normale Supérieure, Paris, France; 2 Ecole Normale Supérieure, IFR36, Plate-forme Transcriptome, Paris, France; Purdue University, United States of America

## Abstract

**Background:**

Diatoms are largely responsible for production of biogenic silica in the global ocean. However, in surface seawater, Si(OH)_4_ can be a major limiting factor for diatom productivity. Analyzing at the global scale the genes networks involved in Si transport and metabolism is critical in order to elucidate Si biomineralization, and to understand diatoms contribution to biogeochemical cycles.

**Methodology/Principal Findings:**

Using whole genome expression analyses we evaluated the transcriptional response to Si availability for the model species *Phaeodactylum tricornutum*. Among the differentially regulated genes we found genes involved in glutamine-nitrogen pathways, encoding putative extracellular matrix components, or involved in iron regulation. Some of these compounds may be good candidates for intracellular intermediates involved in silicic acid storage and/or intracellular transport, which are very important processes that remain mysterious in diatoms. Expression analyses and localization studies gave the first picture of the spatial distribution of a silicic acid transporter in a diatom model species, and support the existence of transcriptional and post-transcriptional regulations.

**Conclusions/Significance:**

Our global analyses revealed that about one fourth of the differentially expressed genes are organized in clusters, underlying a possible evolution of *P. tricornutum* genome, and perhaps other pennate diatoms, toward a better optimization of its response to variable environmental stimuli. High fitness and adaptation of diatoms to various Si levels in marine environments might arise in part by global regulations from gene (expression level) to genomic (organization in clusters, dosage compensation by gene duplication), and by post-transcriptional regulation and spatial distribution of SIT proteins.

## Introduction

In the marine environment, several classes of photosynthetic organisms, including Bacillariophyceae, Chrysophyceae, Silicoflagellates, Prasinophyceae and Radiolarians, can accumulate silicic acid (Si) and/or are capable of creating a silicon-based extracellular skeleton [1], [2], [3], [4], [5]. For some of these microalgae, the availability and distribution of silicic acid is important since it can be a limiting factor that can affect their population dynamics in oceanic ecosystems [6], [7], [8]. For example, most diatoms, which are important components of the phytoplankton community, have an obligate silicon requirement for growth [9], [10], and the silicic acid concentration can limit their growth [11], [12], [13]. However, in spite of many studies on diatoms, little information exists on the genes involved in silicon assimilation or storage.

For diatoms the detailed mechanisms of silicon uptake, storage, targeting and organized polycondensation processes are not completely understood although key factors have been identified. For transport, specific membrane proteins, named the silicic acid transporters (SITs), have been isolated and characterized in a number of diatom species [14], [15], [16], [17], [18], [19]. Interestingly, SIT homologs have also been found in Chrysophyceae [20], [21], [22]. Biochemical and genetic approaches have allowed the identification of a number of long-chain polyamines and proteins that display *in vitro* the ability to create and structure silica particles [23], [24], [25], [26], [27]. Isolation and identification of an enriched cell wall fraction allowed the discovery of a large set of proteins potentially involved in the formation of the frustule in the model centric species *Thalassiosira pseudonana* [28], for which the complete genome is available [29]. More recently, whole-genome expression profile analyses have revealed putative genes involved in silicon bioprocesses [30].

*Phaeodactylum tricornutum* is another important diatom species because it is the first pennate diatom for which the complete genome information is available [31] and molecular tools exist [32], [33], [34]; opening up the possibility to investigate by global approaches the Si-regulated pathways in this clade. In addition, this species is also interesting because of its pleiomorphism with the existence of three morphotypes: oval, fusiform and triradiate [35]. However, silicification is essentially restricted to one valve of the oval cells [36], [37], [38], [39], [40]. For this species we expect that Si-regulated genes could present interesting features because even if all morphotypes assimilate Si [19], [35], [41], [42], [43], there is no Si-requirement for growth [37]. Such properties differ to the situation found in most diatoms where it was shown that the availability of silicic acid is of major influence to cell cycle progression, and that Si deficiency leads to arrests at G1/S or G2/M transition [9], [10]. Thus, it can be envision that *P. tricornutum* should allow the identification of genes that are involved in silicon metabolism, with little or less interference with cell cycle progression.

Here, we report the first genome-wide transcriptome analyses in *P. tricornutum*. We have used a *P. tricornutum* fusiform strain that does not make a silicified valve, allowing us to focus on genes putatively involved in silicic acid sensing, acquisition and storage. We found 223 genes regulated by silicic acid availability including: 13 genes upregulated under Si starved conditions as compared to cells grown in silicic acid supplemented medium (*i.e*., complete medium), and 210 upregulated in complete medium compared to Si starved conditions. We identified several genes potentially involved in specific metabolic pathways such as glutamine-nitrogen, signal transduction or iron regulation processes, and a number of genes encoding putative matrix proteins. We also found that about 25% of the Si-dependent regulated genes are organized in clusters. Among them, a recent tandem duplication of a SIT transporter, named PtSIT2, was verified and the differential expression of this gene was confirmed by qRT-PCRs for both oval and fusiform morphotypes. Localization studies of PtSIT2-GFP showed that this protein is located mainly at the plasma membrane, and in dynamic intracellular vesicles. Moreover, expression studies suggested the existence of post-transcriptional regulations of this transporter. Altogether our data give new support to the hypothesis of a multilevel regulation of SIT transporters.

## Results

### Identification of the silicic acid sensing genes

In order to identify *P. tricornutum* genes that are differentially expressed in presence or absence of silicic acid, we developed an oligonucleotide-based microarray representing the whole set of the identified ORFs. In total, the full-genome array covered about 98% (10,201 out of 10,402 genes) of the nuclear, 85% (34 out of 40) of the mitochondrial, and 98% (132 out of 135) of the chloroplastic protein-encoding ORFs. The resulting arrays were hybridized with cDNAs derived from total RNAs extracted from cells in exponential growth phase cultured for at least three weeks in the absence or the presence of silicic acid (see Materials and Methods). Three biological replicates were hybridized using dye-swap procedures and a total of six arrays were used for statistical analyses. After normalization and data pretreatment (see Materials and Methods) genes for which at least three measurements were available were analyzed for expression changes between replete and deplete conditions using statistical analyses based on the SAM method [44]. This procedure combined with an additional cut-off (mean |log2(ratio)| >1.8) allowed us to retrieve 568 differentially expressed probes. Since some genes could have several probes, in total 223 genes (from the 568 probes) were shown to be differentially regulated, among which 13 genes were upregulated in the Si starved samples as compared to *P. tricornutum* grown in complete medium, and 210 were upregulated in the presence of silicic acid (Supplementary Table S1). QRT-PCR was used to test differential expression of 26 genes (11% of the differentially regulated set). In this analysis 25 of the 26 tested genes were confirmed as differentially expressed (Table 1).

**Table 1:** Quantitative RT-PCR of 26 different genes.

Phaeo JGI Protein ID	Forward (5′-3′)	Reverse (5′-3′)	ΔCT with Si §	ΔCT Si-free §	Fold-change	P-value
p23423/p55090	TGCTGAGCACGCGCCACCGGCC	CCAAATGCGGTGTACTTCTTAC	2.62±0.44	0.22±0.51	0.19	<0.0001
p49598	CGCCGAGTTGGAAATACAAT	TTCCTGTAAGTGGGGGTCAG	4.78±0.42	2.83±1.01	0.26	<0.0001
p14401	ATGGCAGACGAAATTCTTGG	ATTGCACGTTGGATGCAATA	8.42±0.60	6.82±0.68	0.33	<0.0001
p32401	GTCAACCGTCCTTTGGAAGA	ACCGAAACTGGTGACACCTC	5.10±0.42	3.72±0.46	0.38	<0.0001
p45011	AGAAGCACTCGCATTGTCCT	TAAACGGGGCTGTCACTTTC	7.66±0.37	6.43±0.41	0.43	<0.0001
*p48685*	*AGCACTTTGATGCCTGGAAC*	*CACTGGAGTGGGGTCAAAGT*	*5.43±0.26*	*5.25±0.66*	*0.89*	*0.544*
p44994	GAGGTACCGTCACTGCCATT	CGGAGGACACGATTCTTGAT	7.05±0.39	7.97±0.79	1.89	0.011
p52619	GCTGGACCTTCACTCTCGTC	CCTTCATGGTATCGGAGCAT	4.98±0.35	5.97±0.81	1.99	0.007
p8537	TCCGCCTAGTAGCGGTTTAG	CGCCTTGAATATCCACCAAC	13.10±0.79	14.17±1.10	2.10	0.052
p7208	TTTGCACAACTCCTCACTGC	ATTCCTTTGGGCTGTCCTTT	3.55±0.31	4.73±0.31	2.26	<0.0001
p51092	AGGAAGGTGGCCTTGAAGTT	TCAGGCGAAGTTCGTTACCT	6.11±0.76	7.39±0.96	2.43	0.01
p20424	CCGTTACTGGATGGCTTGTT	GTAGTCCTGGGGATCGATGA	1.48±0.51	2.84±1.06	2.56	0.019
p47298	GTATCGCTCGCTTGGAAGTC	CAAGACGTAAAAGGGGTCCA	6.38±0.75	7.77±1.25	2.61	0.023
p47709	GTGCTGGACGAAGTGACAGA	GGGGTTGTTTTGGTACATGG	5.54±0.36	6.96±0.76	2.67	<0.0001
p37052	GAAACTTCAATTCGGGTCCA	TCGAAAAACAGGGGCATAAC	5.05±0.16	6.49±0.71	2.71	<0.0001
p22357	TTGACTTGCTCAACCAGCAC	ATGGTTTCGAGACGCAGAAC	3.83±0.45	5.28±1.28	2.74	0.009
p4823	GCAACTTTGTGGATGTGGTG	ATTTTGTCGAGTTCCGGATG	15.03±0.48	17.08±0.36	4.16	<0.0001
p44523	AATAAGCCTGTCGACGGAGA	AGCTTCACGTACGGCTTTGT	7.96±0.22	10.31±0.74	5.10	<0.0001
p27877	ATTGGAGTCGTTGGAGGTTG	CTAGGACACCCCAGAAACCA	3.22±0.68	5.48±1.47	6.12	<0.0001
p44995	CGAGCCTGAAGAGGAGTGTC	CGTCCTCGGGTATCTACCAA	5.92±0.17	9.33±0.56	10.67	<0.0001
p54986	AGAGGTCGGAAATGTGATGG	GCATAGATGCGCAACTCAAA	4.16±0.55	8.18±0.32	16.16	<0.0001
p44524	GTCGGTACTTTGCCATTCGT	TTGATGAGCCATCTCTGCAC	6.81±0.21	11.12±0.74	19.84	<0.0001
p23658	CAGCTATCACCAACGGGTTT	AATGTACTCGGCGACTTGCT	1.58±1.39	7.45±0.44	58.30	<0.0001
p52498	CTCCCCAAGACACGATCACT	TTGGCCAAGACCGATAAGAC	2.61±0.62	8.65±0.44	65.74	<0.0001
p51183	GAACCTTTACAACGCCCTGA	AAGGACCGTCCACAAAACTG	2.09±0.61	8.39±0.56	78.81	<0.0001
p54987	CTCCCATGGATGTCGAGTCT	CATCCAGTTTTCTGGGCATT	3.51±1.10	11.88±0.56	329.77	<0.0001

§Relative expression (ΔCT relative to the control gene Histone H4) under Si-Free or 350 µM Si. Each value corresponds to the mean from three biological samples performed in duplicates. Significant changes are indicated by a P value <0.05 (Z-test).

**Figure 1:**
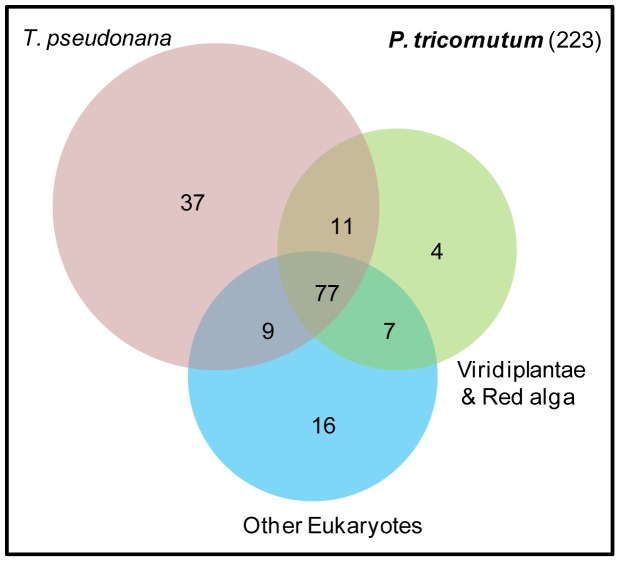
Venn diagram showing how many of the silicic acid regulated genes identified in *P. tricornutum* are also found in some other lineages. Representation of shared/unique Si-sensing genes in *P. tricornutum*, *T. pseudonana*, Viridiplantae (*i.e.*, plants and green algae) & red algae, and other Eukaryotes (*i.e.*, other Chromalveolates and Opisthokonta and Alveolates).

For all of the 223 differentially expressed genes in total, in complement to the information available at the genome browser, we manually edited some of the gene models from the genome browser to get the putative full length gene model, and further searched for InterPro domains (see Materials and Methods and Supplementary Table S2). Using comparative tools, previously developed during the annotation of *P. tricornutum* genome [31], we found that among the differentially expressed genes 59.2% are shared (using as criteria: over 50% amino acid coverage and a cutoff of e<1–05) with the centric diatom *T. pseudonana*, 43.5% with Streptophytes, Chlorophytes and a red algae, and 46.6% with Opistokonts (Fig. 1). Notably, 72.1% of the genes with unknown function are unique to the two diatoms and 44.3% are unique to *P. tricornutum* (*i.e*., not found in *T. pseudonana* genome or in the 21 other eukaryotic full-genome species), and could correspond to diatom genes involved in response to silicic acid, or to the facultative adaptation to silicic acid, respectively.

Among the genes that were upregulated in complete medium (*i.e*., with silicic acid) as compared to Si starved growth condition, we found several genes involved in carbohydrate metabolism pathways such as glycolytic enzymes (p41423 and p54920). Among this set of genes overexpressed in presence of silicic acid, we identified genes encoding for enzymes involved in glutamate pathway (p47298, p51920, p22357; KEGG pathway ref 00251) or metabolism of amino groups (p55010, p25521; KEGG pathway ref 00220) (Supplementary Tables S1 and S2). These pathways are linked to synthesis and metabolic pathways for S-Adenosyl-L-Methionine [45]. Interestingly an S-adenosylhomocysteine hydrolase (p25521), and a choline dehydrogenase (p43604) are also upregulated. In addition, two transporters (p2024 and p768) involved in urea acquisition were also over-expressed in complete medium. Among the other genes overexpressed in the presence of silicic acid, we found several genes encoding putative glycosylated matrix proteins (p48425, p38600, p54178), as well as proteins similar to fibrocystin (p46563, p46559, p46560, p48753) or similar to other extracellular proteins such as elicitin, mucin, flagellin, hrc (p44995, p44994, p47315, p8537, p8524, p51761, p11793, p46346 …). Consistently, several genes involved in protein glycosylation (p47114, p36648, p50429, p40022) were also found to be overexpressed. Interestingly, a number of proteins putatively involved in various intracellular signaling or post-transcriptional regulation processes were upregulated in presence of silicic acid such as guanylate cyclases (p38814, p47709, p4823, p48678, p50428, p49555), kinases (p43087, p8877, p16940, p34085, p54760, p54547), a hydroxylase involved in calcium homeostasis (p48622), proteins involved in vesicular trafficking (p44524, p45476) or in regulation processes such as HSF (p34415, p37052) or transcription regulators (p51183, p8282, p47669, p45280) (Supplementary Table S1).

Among the genes that were found to be upregulated under silicic acid starvation we identified genes involved in carbon acquisition (p32401), glyoxylate cycle (p14401), a RING finger ligase involved in ubiquitination (p45011), and several transporters (p55090 and p23423) (Supplementary Table S1).

### Organization in clusters of the differentially expressed genes

**Figure 2:**
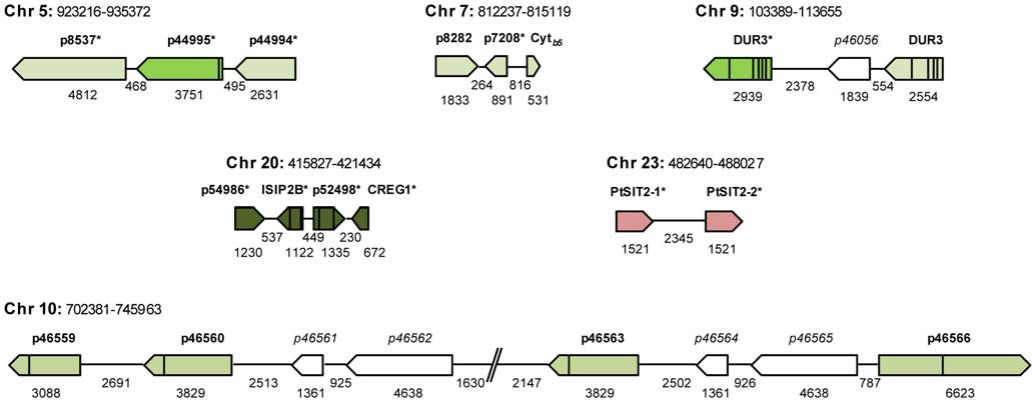
Examples of Si-regulated genes organized in clusters or found in the same vicinity. All non-italicized green or red fonts depict gene products found to be upregulated in silicic acid supplemented medium compared to cells grown in Si-starved conditions or genes upregulated in Si starved conditions, respectively. The star indicates the genes for which the differential expression was verified by qRT-PCR (associated data are presented in Table 1). The interruption in Chr 10 corresponds to a stretch of about one hundred unsequenced nucleotides.

The mapping of the differentially expressed genes on the scaffolds/chromosomes revealed the presence of clusters of expression. Thus, we identified 20 clusters of two genes (17.9% of the total number of the differentially expressed genes), 3 clusters of 3 genes (4.0%) and 1 cluster regrouping 4 genes (1.8%) (Supplementary Table S1 and Fig. 2). Moreover, when we considered genes clustered at the same locus with presence of maximum three non regulated between two differentially expressed genes, the percentage of clustered genes raze up to 30% (Supplementary Table S1). Among these “clusters” some of them correspond to genes with a similar function such as the sodium solute symporters (p2024-X-p768) (Fig. 2), putative helicases (p36749-X-X-p13230), or genes encoding putative matrix proteins (p8537-p44995-p44994, p51761-p11472-X-p11401, p6559-p46560-X-X-p46563). The largest cluster identified (p54986-p54987-p52498-p51183), groups a putative transcription factor, with two putative cell surface proteins and an encoded protein induced under iron starvation (Fig. 2). Interestingly, this latest cluster corresponds to the genes that present the highest expression variation between our two studied conditions (with confirmed qRT-PCRs folds >16) (Table 1).

Interestingly, we found that some of the genes that are organized in clusters may reflect recent duplication events. Here we identified, the two the putative extracellular matrix protein p44994 and p44995 share 81% overall identity in their coding region (Fig. 2), the two DUR3 genes (p2024 and p768) present an overall identity of 84% in their coding region (Fig. 2), and three genes in the chromosome 10 (p46560, p46561 and p46562) were found to present 99% overall identity with (p46563, p46564 and p46565) (Fig. 2). We also found that two of the most strongly expressed genes under Si-starvation, p23423 and p55090, correspond to a cluster of two genes, named PtSIT2-1 and PtSIT2-2, encoding putative silicic acid transporters (Fig. 2). The two *PtSIT2* genes were arranged in tandem, separated by a distance of 2.3 kb from *PtSIT2-1* stop codon to *PtSIT2-2* start codon (Supplementary Fig. S1).

In order to check whether such *PtSIT2* cluster organization was not due to genome sequence misassembly we performed different amplifications by PCR using genomic DNA (Supplementary Fig. S1). Amplifications from gene to gene, from intergenic region to gene, as well as from upstream and downstream regions confirmed the PtSIT2 duplication. Sequence analysis revealed that for *PTSIT2-1* and *PtSIT2-2*, the coding region was 100% conserved at the nucleotide level, as well as 0.44 kb and 0.2 kb of the 5- and 3′-UTR region, respectively. These observations suggest a recent and local duplication event in this ecotype.

### Analyses of *Pt*SITs

**Figure 3:**
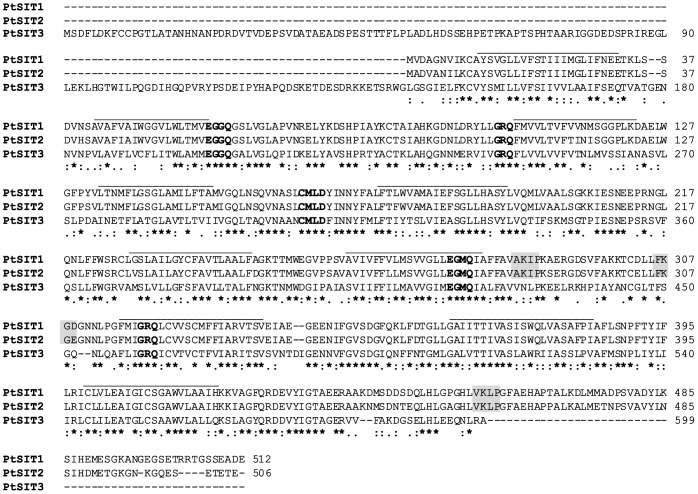
Alignment of the predicted amino acids of PtSITs. The lines correspond to the putative trans-membrane helices predicted using TMPred or HMMTOP. The regions in bold correspond to amino acids proposed to be involved in Si(OH)_4_ transport, and in grey to putative motif for sumoylation. The alignment was performed with CLUSTAL-W run with default multiple alignment parameters.

To elucidate silicic acid acquisition in *P. tricornutum* we further characterized the SIT gene family. Genome analyses allowed recovery of four different *PtSITs*, named *PtSIT1*, *PtSIT2-1*, *PtSIT2-2* and *PtSIT3*, located on chromosome 18, Chr 23, Chr 23 and Chr 5, respectively. Each of the *P. tricornutum SIT* genes encodes an uninterrupted open reading frame. We also noticed that the *PtSIT3* ORF contains two putative start codons: one being located at the very beginning of the region presenting homology with the other SIT genes and an additional upstream ATG in the *PtSIT3* transcript (Fig. 3). We then performed 5′-RACE PCR experiments for each of the *PtSIT* genes (see Materials and Methods). These experiments confirmed the existence of a 5′ extension of the *PtSIT3* transcripts compared to the two other *PtSITs*.

The amino acid sequence of full-length PtSIT1 (the JGI protein ID is p48707), PtSIT2 (p23423 and p55090) and PtSIT3 (p1451) were then aligned using ClustalW (Fig. 3). As previously described for other SIT proteins, analyses of PtSITS protein sequence using TMPred or HMMTOP program predicted 10 putative trans-membrane segments (TMS) (Fig. 3). The previously proposed GXQ silicic acid binding sites located in transmembrane segments 7 and 8 [18], and the conserved CMLD motif located between transmembrane segments 4 and 5, proposed to be involved in Si capture via Zn^2+^ ion binding were present in all PtSITs (Fig. 3). Pairwise analyses revealed that the identity and similarity between PtSIT1 and PtSIT2 are about 90% and 95%, respectively (Table 2). The identity drops to about 50% when PtSIT3 is now compared to either PtSIT1 or PtSIT2 (Table 2). Independently of the method used (either approximate methods or maximum likelihood methods; [46], [47]) analyses revealed that the non synonymous substitution rates (Ka) was about 10-fold higher for PtSIT3 compared to PtSIT1 and PtSIT2, and that the synonymous substitution rates (Ks) was about 3-fold higher (Table 2).

**Table 2:** Non-synonymous (Ka) and synonymous (Ks) substitution rates of PtSITs.

Gene or Protein	Ka	Ks	Ka/Ks	Length (nt)	Identity (%)	Similarity (%)
**PtSIT1/PtSIT2**	0.051	1.40	0.036	1518	90	95
**PtSIT1/PtSIT3**	0.415	2.60	0.159	1362	51	70
**PtSIT2/PtSIT3**	0.420	2.67	0.157	1362	50	70

**Figure 4:**
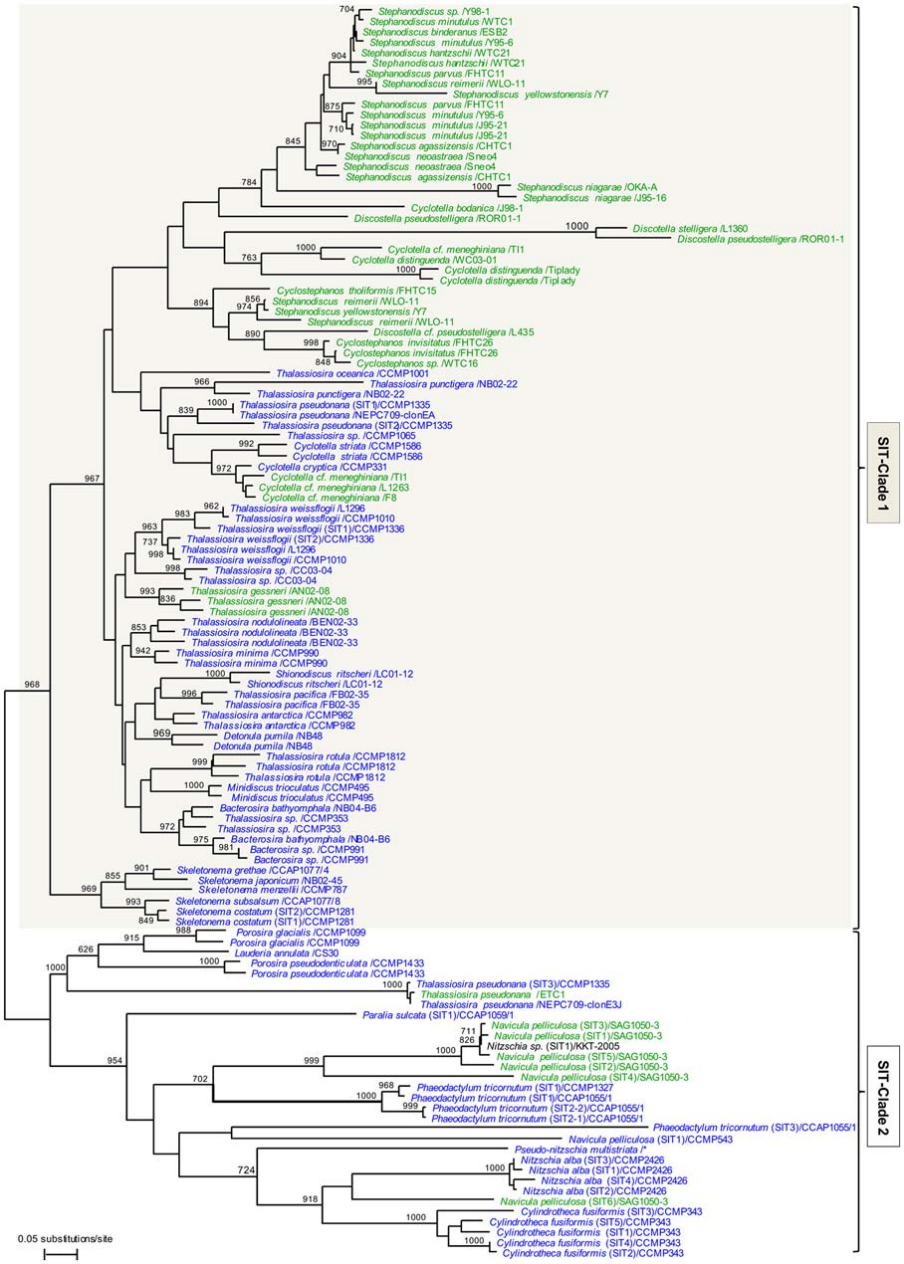
Phylogenetic tree of SIT amino acid sequences. The tree was inferred based on neighborg-joining from a distance matrix, considering 230 aa positions from 121 SIT sequences. Each SIT sequence is identified by the name of the cultured strain from which the DNA was sequenced (for one diatom the origin of the strain could not be retrieved), when available the SIT gene name is indicated. The origin of the diatom strains is presented as marine (blue), freshwaters (green) or unknown (black). The numbers at the nodes correspond to the calculated Bootstrap values (out of 1000 resamplings). The scale bar indicates 0.05 fixed amino acid substitution per site.

**Figure 5:**
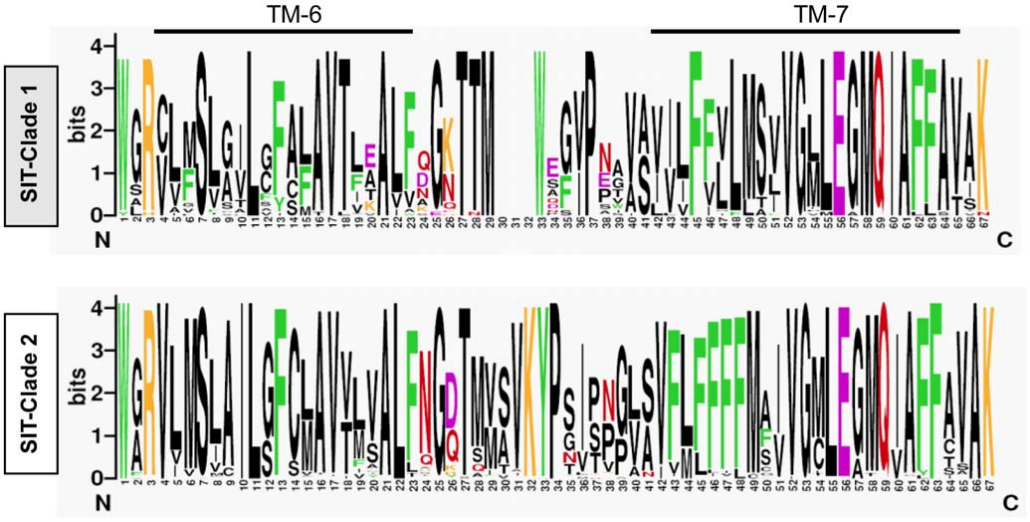
Logos of the region encompassing the putative trans-membrane domains 6 and 7 of SITs. The divergence in the amino acid composition of the extra-membrane region between TMS 6 and TMS 7 is sufficient to define two Clades (also see Fig. 4). Clade 1 corresponds to most of the SIT sequences from centric diatoms, and the Clade 2 includes all the known sequences from the pennates and some sequences from the Thalassiosirales. The color code used is: purple for amino acids with an acidic lateral chain, orange for basic, green for aromatic, red for asparagine and glutamine and black for the rest. The data used to generate the Clade 1 and Clade 2 Logos, consists of alignments of 92 and 34 protein sequences, respectively.

To built up a phylogeny of *P. tricornutum* SITs, we used a large number of sequences from centric diatoms (from both fresh and marine waters) that have recently been made available [14] along with previously reported sequences [15], [17], [18] and a partial sequence from the toxic diatom *Pseudo-Niztschia multistriata*. Global analyses confirmed that the silicic acid transporters can be divided into two clades (see also [14], [18]): clade 1 contains sequences from centric diatoms and clade 2 contains sequences of centrics and all the available pennates (Fig. 4). Interestingly, further manual examination of the protein sequences allowed the identification of a minimal region between TMS-6 and TMS-7 which is sufficient to differentiate between these two clades (Fig. 5).

### PtSIT expression in various morphotypes

To analyze the influence of the morphotypes on the expression of the *PtSITs* genes, we performed qRT-PCRs for the strains named Pt1 (*ca.* 99% fusiform morphotype) or the strains named Pt0 (*ca.* 95% oval morphotype) (see Material and Methods and [40]). However, to avoid cross amplification we first cloned each of the *PtSIT* genes, and then verified the specificity of the primers used to amplify only a single SIT gene (see Materials and Methods). Statistical analyses of the results of qRT-PCRs performed in duplicates for eight biological samples, from strain grown with or without silicic acid, confirmed that *PtSIT2* was upregulated by a factor of 3.72±0.008 and 2.60±0.003 in Si-free medium for both fusiform and oval morphotypes, respectively (Table 3). Such data confirmed the results obtained by microarray experiments. Surprisingly, *PtSIT1* was significantly upregulated only in fusiform cells (strain Pt1) whereas *PtSIT3* was not significantly expressed in either of the two strains analyzed (Table 3).

**Table 3:** Relative expression level of PtSITs.

Cell Morphology	Fusiform	Fusiform	Fusiform	Oval	Oval	Oval
Gene	PtSIT1	PtSIT2	PtSIT3	PtSIT1	PtSIT2	PtSIT3
**Fold induction SP/SM**	−2.70	−3.72	−1.49	−0.75	−2.60	−1.17
Z-test	0.027	0.008	0.480	0.487	0.003	0.717

Each value corresponds to the mean from eight biological samples performed in duplicates from cells grown in absence (SM) or presence (SP) of 175 µM silicic acid. Significant changes are indicated by a P value <0.05 (Z-test).

### Cellular localization and protein level regulation of PtSIT2

**Figure 6:**
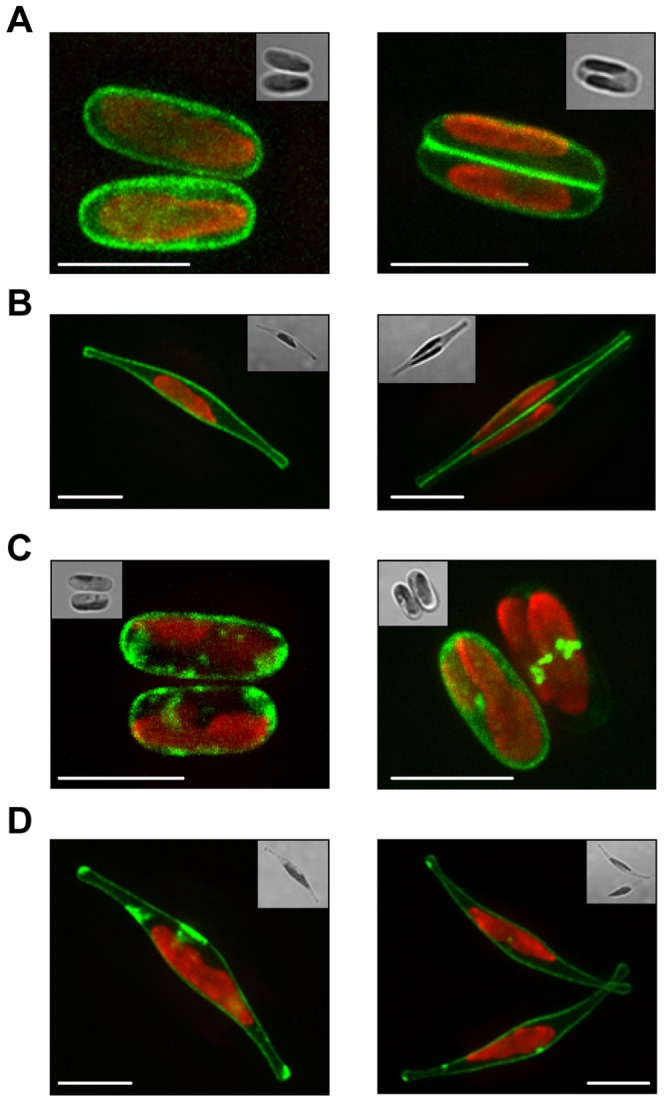
Localization of PtSIT2-GFP. A. Maximum projected stacks reveal the localization of PtSIT2-GFP at the cytoplasmic membrane in oval cells. B. The localization of the PtSIT2-GFP in fusiform cells. The higher fluorescence intensity seen at the interface between daughter cells, presumably account for the addition of the signals belonging to the adjacent plasma membranes of the dividing cells. C. In the absence and more often in the presence of silicic acid the signal corresponding to PtSIT2-GFP was found as patches on the cell membrane or in intracellular loci. Such localization could correspond to intracellular vesicles or degradation sites, and to membrane trafficking vesicle. D. Same as in C but for fusiform cells. The GFP signal is in green, the auto-fluorescence (chloroplast) is in red, and the insert to the Bright field image. Before reconstruction, the fluorescence micrographs were enhanced by digital deconvolution resulting in improved signal-to-noise ratio and resolution. The scale bar corresponds to 5 µm.

**Figure 7:**
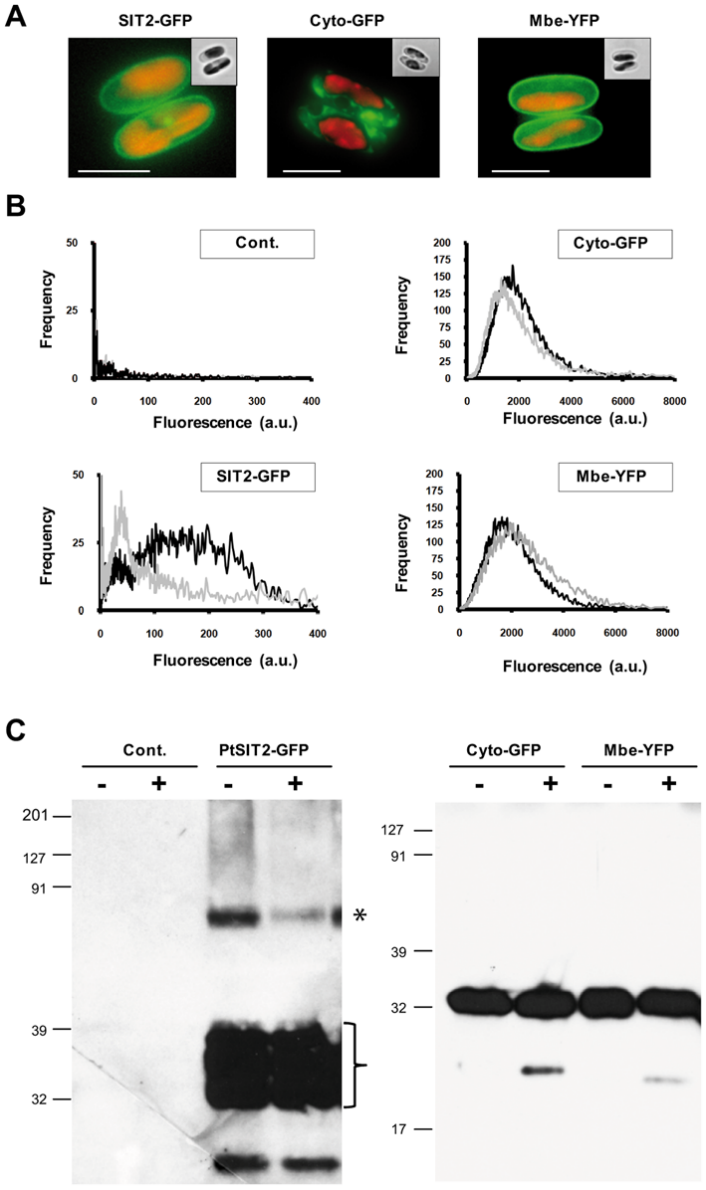
Expression of PtSIT2-GFP. A. Fluorescence images of transgenic cells expressing under the same *fcpA* promoter the fusion proteins PtSIT2-GFP, a Cyto-GFP, or a Mbe-YFP proteins. B. Cells expressing the fusion proteins were analyzed by FACS. The relative fluorescence intensity recorded in the green channel corresponds to the GFP (*left panel*), and in the red channel to the autofluorescence (*red channel*). Cells were grown in the absence (*black line*) or in the presence of silicic acid (*grey line*). The control (*Cont*.) corresponds to *P. tricornutum* cells untransformed. C. Total protein fractions from the different cultures grown without (−) or with (+) silicic acid control or transgenic cells expressing PtSIT2-GFP, Cyto-GFP or Mbe-YFP were separated on Tris-acrylamide gel and GFP was detected using a polyclonal antibody. Note the higher amount of full length SIT2-GFP product (*star*) for cell grown in the absence of silicic acid, and the accumulation of degradation products for the SIT2-GFP. The exposure time for the left and right panel is different since the Cyto-GFP and Mbe-YFP proteins expression level were higher than the one of the full-length SIT2-GFP. The scale bar corresponds to 5 µm.

The observed duplication of *PtSIT2*, together with its upregulation in silica starvation condition, led us to further investigate its expression and localization. We fused *PtSIT2* with a fluorescent reporter (GFP) and cloned the translational fusion, in a diatom expression vector containing the *fcpA* promoter (see Materials and Methods). To visualize the localization of the silicic acid transporter in both oval and fusiform cells the new plasmid, named pPtSIT2-GFP, was used to transform the Pt0 strain and GFP fluorescence was analyzed. After transformation and selection for transgenic cell-line, epifluorescence microscope studies showed the first localization of a SIT transporter in diatoms, with Sit2-GFP localized at the plasma membrane in both oval and fusiform cells (Fig. 6A–B). Such localization of a silicic acid transporter was also clearly visible in dividing cells (Fig. 6A–B). We also noticed the accumulation of PtSIT2-GFP in intracellular vesicles and as patches at the plasma membrane (Fig. 6C–D). As control, a vector containing under the *fcpA* promoter GFP without the *PtSIT*2 gene (Cyto-GFP) or GFP fused to a membrane localization signal (Mbe-GFP), was used to transform Pt0 cells. The presence of vesicles expressing the GFP was not observed in cells expressing under the same *fcpA* promoter the membrane localized YFP-variant (Mbe-YFP in Fig. 7A) or the GFP alone (Cyto-GFP in Fig. 7A).

To test whether PtSIT2-GFP protein expression levels depend on the availability of silicic acid in the medium, we analyzed the reporter fluorescence intensity by flow-cytometry. Results confirmed that in Si-free medium a subpopulation of cells show a higher GFP fluorescence, whereas cells grown in presence of Si show on average lower signal intensity (Fig. 7B). As a negative control we used an untransformed strain which did not show any GFP signal, and as positive controls we used strains expressing either the Mbe-YFP or the Cyto-GFP. These latest transgenic strains did not show any significant variation of their fluorescent signal independently of the presence or absence of silicic acid in the medium, although all the fusion proteins were expressed under the same promoter (Fig. 7B).

Finally, we performed Western blot experiments using an anti-GFP antibody. Although protein degradation was observed, we found that the PtSIT2-GFP full length protein was slightly more abundant in a Si-free compared to a Si-containing medium (Fig. 7C, right panel). In contrast, control experiments showed that under the same conditions, the protein level of a Cyto-GFP fusion or a Mbe-YFP fusion did not show variation (Fig. 7C, left panel). Altogether, our results are consistent with the hypothesis that *PtSIT2* gene is regulated at both transcriptional (*cf*. microarray and qRT-PCR data) and post-transcriptional levels.

## Discussion

### The transcriptomic response to silicic acid availability of *P. tricornutum* fusiform cells

**Figure 8:**
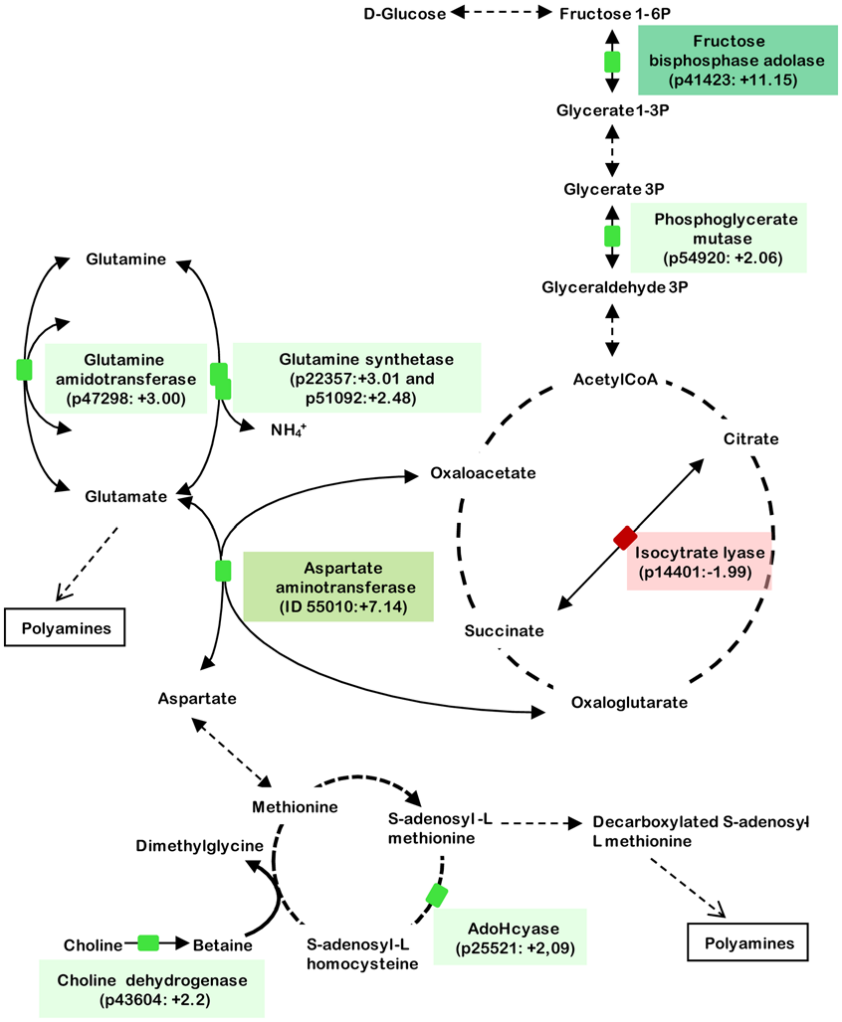
Metabolic pathways associated with silica-regulated genes. Silica-regulated genes revealed by microarray assay were represented together with the enzymatic reactions they catalyze and within associated metabolic pathways. Protein ID, annotation and induction fold are indicated within colored boxes. Differentially regulated genes were highlighted in green or in red for genes upregulated in the presence or absence of silicic acid, respectively. N-X represent a glutamine amidotransferase (class II) substrate where N is a transferred ammonia from carbon-nitrogen group. This reaction may be involved in biosynthesis of purine, glucosamine 6-phosphate or asparagine. Genes involved in carbon metabolism are present, especially within glycolysis (p41423 and p54920). Another group of genes involved in nitrogen metabolism (KEGG ref 00910) are also represented such as enzymes involved in glutamate pathway (p47298, p51920, p22357; KEGG ref 00251) and metabolism of amino groups (p55010, p25521; KEGG ref 00220). Interestingly glutamate and aspartate are precursors of ornithine and methionine, that are involved in polyamine biosynthesis, where AdoHcyase (p25521) and Choline dehydrogenase (p43604) are involved.

The potential of microarray technique was used to analyze the global response of *P. tricornutum* fusiform cells to the availability of silicic acid. Among the 210 genes that were found to be over-represented in the presence of silicic acid we identified several genes encoding for enzymes involved in glutamine/glutamate metabolism (Fig. 8). Interestingly, a previous study on *Thalassiosia pseudonana* has shown that a glutamate acetyltransferase was involved in Si metabolism [28]. We also found genes coding for transporters of metabolites related to nitrogen assimilation and transfer. Indeed, an ammonium transporter (p27877) and other nitrogen containing compounds transporters (p20424, p768, p52619) were found to be upregulated in the presence of silicic acid in *P. tricornutum*. Interestingly, an S-Adenosylhomocysteine hydrolase (p25521), as well as a choline dehydrogenase (p43604) were also upregulated in presence of silicic acid (Fig. 8). Theses enzymes are involved in the S-adenosyl-methionine (SAM) cycle, which participate to methyl or propyl-amine group transfer, and to polyamine synthesis [45]. Choline dehydrogenase upregulation (p43604) may also be involved in osmotic regulation through betaine production. Therefore a possible hypothesis to explain the activation of nitrogenous secondary metabolites pathway in *P. tricornutum* may be that these compounds might be involved in osmotic regulation and ion homeostasis in response to the presence of extra-cellular silicic acid, and/or to the regulation of its intracellular accumulation. Such hypothesis of a modification of the cellular homeostasis in the presence of silicic acid may explain the large variety of transporters that are found over-expressed (p54522, p13078, p49842, p50026). Interestingly, SAM cycle also contributes to polyamine biosynthesis (Fig. 8), and DUR3 genes (p20424, p768) have been shown to be responsible for transport of polyamines and urea in several organisms including yeast, plants and algae [48], [49], [50].

Moreover, among the genes up-regulated in presence of silicic acid we also found an important number of putative transmembrane proteins that could be glycosylated (p48425, p38600, p54178, p46563, p46559, p46560, p48753, p44995, p44994, p47315, p8537, p8524, p51761, p11793, p46346). Among these proteins some have striking lysine-arginine-serine-rich (KRS-rich) repeats (p44680, p41599) (Supplementary Table S1).KRS-rich repeats were identified in a silaffin gene from the pennate *Cylindrotheca fusiformis*, and demonstrated to be involved in silica polycondensation [51], [52]. Among the *P. tricornutum* proteins that present KRS-rich repeats we noticed the presence of Proline Threonine repeats (PT-repeats) region in several of them (p47315, p8524, p51761, p11401, p11472) (Supplementary Table S1). The presence of PT-repeats was described for several *Thalassiosira pseudonana* silaffins [53]. Even though our results were obtained from fusiform cells, it can not be ruled out that these genes might be involved silicification bioprocesses since silica fiber-like structures have been identified within the organic part of the three *P. tricornutum* morphotypes [36]. Alternatively, transmembrane KRS-rich or glycosylated proteins together with the polyamine biosynthetic pathway may be involved in intracellular silicic acid storage. Consistently, previous studies in *T. pseudonana* have shown that several extracellular matrix proteins presenting similarities with extracellular matrix components are upregulated in the presence of silicic acid [28].

Interestingly, among the genes differentially expressed in the presence of silicic acid we identified six genes that present an adenylyl/guanylyl cyclase domain (Supplementary Table S1). A survey of the complete *P. tricornutum* genome revealed a total of 14 encoded proteins that present this domain. The upregulation of these genes suggests that cyclic nucleotides could be involved in silicic acid signaling in *P. tricornutum*. Regulation of cyclic nucleotides has been rarely explored in diatoms, and only scarce studies exist. It was shown that cAMP level rises during recovery from Si-starvation and that the activity of a cyclic nucleotide phosphodiesterase increases upon addition of silicic acid in *C. fusiformis* [54], [55]. More recently, it was shown that cytosolic cAMP can regulates CO_2_-acquisition system by regulating the expression of a gene encoding a beta carbonic anhydrase [56]. It was also shown that in *P. tricornutum* although change in the intracellular calcium concentration was observed for different stimuli (including Fe, osmotic stress and fluid motion), several ions such as silicic acid, nitrogen, or phosphate did not induce calcium signaling [57]. Combined with these earlier studies, our results allow us to propose the hypothesis that the response of marine diatoms to [C0_2_] and/or [Si] might be controlled by specific receptors and feedback mechanisms that could be mediated by the same second messenger, and that this messenger could be cAMP.

We then compared our data on the global Si-response to other large scale analyses, including a recent analysis on iron limiting conditions in *P. tricornutum* [58]. Interestingly, we found that about 17% (38 out of 223) of Si-sensitive genes were also varying in Fe-limited conditions (Supplementary Table S1). The common genes are involved in a few metabolic pathways including light perception, or correspond to specific genes found to be induced under iron limitation. For example, two genes involved in pigment metabolism, a fucoxanthin-chlorophyll a/c-binding protein (p54065) and a flavodoxin (p23658) were upregulated in Fe-starved or Si-acclimated *P. tricornutum* cultures (Supplementary Table S1). These observations suggest that Fe and Si could be involved in the adjustment of the general metabolism including the one of the photosynthetic apparatus. Moreover, we found that the most upregulated gene cluster under iron starvation (p54986-ISIP2B-p52498-CREG1; Fig. 2) also corresponds to the most expressed cluster in complete medium (Supplementary Table S1). Even if growth rates of *P. tricornutum* fusiform cells are not influenced by Si availability, whereas iron limitation had a major impact on cellular growth, our comparative analyses give new support to the existence of a physiological link between Si and Fe. We also compared our transcriptomic results to a recent study of Si-starved and Fe-starved conditions in the centric diatom *T. pseudonana* [30], but unfortunately only a few genes were found to have the same kind of response in *P. tricornutum* and *T. pseudonana*, and a few were found to respond in opposite ways in the two strains (Supplementary Table S1). However, even if there is no real commonality among the treatments performed in our studies and those of Mock *et al* [30], taken together these results highlight that a link between silicic acid and iron metabolism might be a common feature in different diatom species.

### Regulation of the silicic acid transporters in *P. tricornutum*

Analysis of *P. tricornutum* genome revealed four *PtSIT* genes, located on three different chromosomes, with two duplicated genes on the same chromosome (Fig. 2 and Supplementary Fig. S1). Gene duplication can occur via several mechanisms, including segmental duplication, tandem duplication and retroposition. During evolution duplicate genes are retained because the redundancy conferred by the duplicate genes might facilitate species adaptation and genetic robustness against null mutations [59], [60]. Interestingly, our analysis of the *Ptsit2* cluster suggests that these two genes are the result of a recent duplication event, this duplication might have been facilitated by a *Ty1/copia*-like retroelement localized close to the duplication (Supplementary Fig. S1). Species-specific gene duplication events and segmental duplications were already noticed in diatoms [31], and it has already been proposed that diatom-specific copia-retrotransposable elements may have contributed in the *P. tricornutum* genome to the expansion of diatom specific genes [31]. However, even if the duplication of *PtSIT2* might be advantageous for this ecotype (Pt1) it does not seem to be necessarily linked to the cellular morphotype since Pt1 cells are predominantly fusiform in culture. Moreover, we identified that one of the *PtSIT* genes, *PtSIT3*, shows a high degree of divergence as seen by the large number of nonsynonymous and synonymous substitutions. In addition, the presence a long N-terminal extension suggests that *PtSIT3* might have already become a pseudogene; a result consistent with its low mRNA expression level. Alternatively, it can be proposed that PtSIT3 protein might be involved in a different function that Si-transport, and would therefore be expressed only in certain environmental conditions. Such hypothesis is supported by the detection of PtSIT3 ESTs in libraries from Nitrate-starved and Fe-starved conditions (not shown). Another possibility to explain the divergence of PtSIT3 protein could be that this gene evolved into a specific kind of silicic acid transporter able to transport the deprotonated form of silicic acid (Si(OH)_3_−); a unique feature reported for *P. tricornutum* [41], [43]. The presence of several SIT paralogues has been described in the past, with six genes in *Navicula pelliculosa*, five in *C. fusiformis*, four in *Nitzschia alba*, three in *T. pseudonana*, and at least two copies in a large number of centric and pennate species [14], [18], [61]. Ancient duplication events followed by relaxed purifying selection might explain that SIT genes from centric species could be separated into two different clades (see Figs. 4 and 5, and [14]). The presence of several paralogues appears to be a general feature for diatoms.

Previous kinetic analyses of *T. pseudonana* SIT transporters showed that protein levels, mRNA, and uptake capacity did not show a strict correlation, suggesting that Si transport is regulated at least at three different levels: transcriptional, posttranscriptional and at the level of protein transport activity [61]. In this study, we have shown that *PtSIT2* gene is regulated at the transcriptional level in both oval and fusiform cells. Moreover, our data are consistent with the existence of post-transcriptionnal regulations of the PtSIT2-GFP construct. In addition, we have shown for the first time that a PtSIT2-GFP fusion protein is localized at the plasma membrane, which might reflect the function of this protein for uptake of Si(OH)_4_. We also noticed intracellular accumulations of the PtSIT2-GFP proteins at locations that could correspond to trafficking vesicles and/or degradation sites. Related to these observations we found that these vesicles seemed to become more important in early response to silicic acid replenishment. Other experiments revealed that the vesicle-like intracellular accumulation PtSIT2-GFP also increases in the presence of the proteasomes and calpains inhibitor MG-132 (not shown). Nonetheless, since PtSIT2 targeting and/or degradation dynamics are also visible in fusiform cells, it is possible that the regulations are not entirely specific to cell cycle progression and to valve synthesis *per se*. Even if more quantitative experiments are needed to reach conclusive results, our data suggests that targeting to the plasma membrane and/or activation of a degradation pathway(s) could be very important mechanisms to regulate the activity of PtSIT2. These result complete the recent revisited model proposed for the regulation of Si transport in diatoms [19], and help to explain why the transport capability are different upon short term or long term starvation. All together our data combined with previous studies, such as for example high level of *TpSIT* mRNA in Si starved cells [30], further demonstrate that the control of SIT-mediated transport capability is an important issue in the global regulation of Si uptake.

## Materials and Methods

### Cell Culture and Image Acquisition

The fusiform strain used for complete genome sequencing is named Pt1 (named Pt18.6, CCAP1055/1) and was also used for microarray development. The other strain used is the oval morphotype, named PT0, is available at the Algae Culture Collection of Göttingen University (strain 1090-1a). Diatom cells were maintained at 19°C under light:dark regime (14 h:10 h) maintained to 75 µE.m^−2^.s^−1^ light intensity. Cells were cultured in polycarbonate bottles in an enriched artificial sea water media [40]. When present, silicic acid was added in the form of Na_2_SiO_3_.9H_2_O (at 350 µM or 175 µM). Soluble Silicic acid concentrations where measured using the blue silicomolybdic assay [62]. Images were obtained with a Leica DM-IRB microscope as previously described [63]. The filter sets used were: for auto-fluorescence, excitation (Ex) at 485/25 nm and emission (Em) at 675/50 nm, and for the green signal was Ex at 485/25 nm and Em at 535/30 nm. All the presented images correspond to Z-D projections.

### Microarray assay

Total RNAs from Pt1 strain grown with or without silicic acid (350 µM) were extracted at mid day from cells in exponential growth phase (*ca.* 2–5.10^6^ cell/ml) as described in [58]. Briefly, RNAs of each independent preparation were reverse-transcribed and labeled with Cy3 or Cy5 dye using the indirect labeling procedure. We then hybridized 1.5 µg of labeled cDNA with GE 4×44K DNA chip manufactured by Agilent (AMADID 015761). The full-genome array covered about 98% (10,201 out of 10,402 genes) of the nuclear, 85% (34 out of 40) of the mitochondrial and 98% (132 out of 135) of the chloroplastic protein-encoding ORFs. On the array, 0.56% of the genes are covered by one, 14.76% by two, 9.17% by three, 11.15% by four and 63.35% by five 60-mer oligonucleotides. Each array hybridizations was replicated using dye-swap. Arrays were read using a GenePix 4000B scanner (Molecular Devices, Sunnyvale, CA, USA) and the signal segmentation was done using the GenePix Pro 6.1 software (Axon). Data pretreatment was applied on each result file to discard GenePix flag and saturating spots. The data were normalized without background subtraction by the global Lowess method performed with the Goulphar software [64]. An expression matrix was created gathering all normalized data for each comparison. The matrix was pre-treated to filter out expression profiles with missing values. Finally, differential analysis was performed using Significance Analysis of Microarrays [44], for each comparison setting the FDR (median) at 0.08%. The complete microarray data and the related protocols are available at the GEO web site (www.ncbi.nlm.nih.gov/geo/), accession number: GSE12015.

### Genomic information and sequence analysis

The genomic information results from the complete sequencing of the genome of *P. tricornutum* strain CCAP1055/1, and is available at http://genome.jgi-psf.org/Phatr2/Phatr2.home.html. For the identity and similarity calculation we used wBlast2. The 223 predicted proteins corresponding to the upregulated transcripts were used for the further comparative analysis against different taxonomical lineages. For the gene family analysis a similarity search was performed (all-against-all BLASTP; E-value cutoff 1.E-5) using the predicted protein sequences of the two diatoms and of 21 other species as described in [31]. GO and InterProScan homology (E-value cutoff, 1.10^−3^; lowest percentage identification, 29) annotations were performed with the online version of the Blast2GO v2.3.1 program [65]. However, some gene models from the Phatr2 have to be corrected. Therefore searches for protein domains were also performed manually using InterProScan and PBlast. Sequences of the *PtSITs* can be retrieved from GenBank using the accession number: PtSIT1 (EU879093), PtSIT2-1 (EU879094), PtSIT2-1 (EU879095) and PtSIT3 (EU879096). The partial sequence of the diatom *Pseudo-Niztschia multistriata* is derived from ESTs analyses (Alexander Luedeking, personal communication). The other SIT sequences used are accessible from GenBank. To construct phylogeny, sequences were first aligned with ClustalW, edited in BioEdit to remove gaps, and then Neighbor-joining trees were constructed and drawn with TREECON for windows. Ka and Ks values were calculated using a recently developed model-selected method based on maximum likelihood [47]. Putative sumoylation sites were identified using SUMOplot^TM^, using a cutoff of 0.65.

### PCR assays

Quantitative PCR was performed using an ABI 7900 machine and Eurogentec SYBR green I MasterMix Plus on equal amounts of total RNAs. Reverse transcription was performed on 1.5 µg of total RNA (extracted according to the protocol described in [58]) using Random Primer and Superscript III (Invitrogen). The RACE-PCR experiment was performed with SMART RACE cDNA Amplification Kit (Clontech) according to the manufacturer protocol and using 0.5 µg of total RNAs extracted from PT1 cells grown in the presence of silicic acid. To avoid cross amplification we first cloned the individual genes into a generic vector and verified by PCR that the designed primers amplify only the corresponding genes. The specific oligonucleotide used are (5′-TGCATACCCTCGAGCAAACCAACAAC-3′) and (5′-TGACATGTTGACAACAAAGACAGTCAAC-3′) for *PtSIT1*, (5′-TGCATACCCTCGAGCAAACCAACAAC-3′) and (5′-TGACATGTTGACAACAAAGAGTCAAG-3′) for *PtSIT2*, and (5′-GGGAGATTCACGACGGCGAAGAGAG-3′) and (5′-GTTTCCTTGGTGGGCGAGTTTGGTG-3′) for *PtSIT3*.

### Plasmids construction and diatom transformation

The SIT genes were amplified using a high fidelity pfx DNA polymerase (Invitrogen) according to the protocol of manufacturer. PCR was performed on purified DNA from PT1 using the upstream (5′-CGGAATTCGACGACACCATGGCGGACGTT-3′) and downstream (5′-CGGGATCCTCGGTCTCAGTCTCTCGGACTC-3′) oligonuclotides containing EcoRI and BamHI restriction sites, respectively. The amplified DNA fragment, which correspond to the ptSIT gene, was first purified and cloned in frame with EGFP gene into a pEGFP-N1 plasmid (Clontech) and then cloned into a pPhaT1 plasmid [66]. In the resulting constructs the transgenic gene is under the control of a *fcpA* promoter (fucoxanthin chlorophyll binding protein A), and the resistant gene *sh ble* for selection is under *fcpB*. The other constructs used correspond to an EGFP gene alone or to a fusion between EYFP gene and a membrane targeting signal were also under the same *fcpA* promoter (for further description of the membrane localized EGFP see [40]). After transformation by tungsten microparticle bombardment at 1550 psi (Bio-Rad Model PDS-1000/He Biolistic Particle Delivery System) the desired transgenic diatoms were replated with 10 µg.ml^−1^ of phleomycin, following described protocols [66].

### Flow cytometry analyses

All flow cytometric analyses were carried out on a Cell Lab Quanta™ SC flow cytometer (Beckman Coulter, CA, USA). A 488-nm laser was used for excitation. Green fluorescence was collected through a (525±30)-nm band-pass filter and red fluorescence was collected through a 670-nm long-pass filter. Data acquisition was done at a low flow rate (*ca*. 5 µl.min^−1^) for 3 to 10 min depending on the concentration of the target population (which varies between 1–5 10^−6^ cell ml^−1^), with a total of 18,000 to 20,000 analyzed cells. Cytograms were analyzed using Cell Lab Quanta software for cell counts, and XLSTAT software (Addinsoft, France) was used for further analyses and figures. Since the promoter that drives the expression of the fluorescent protein is the *fcpA* promoter all experiments were performed at approximately during the same period of the day which correspond to the middle of the light period, and the same cell populations were also verified under a fluorescence microscope.

### Immunoblotting

Equal amount of exponentially growing cells were resuspended in a buffer, containing 6% SDS, 10 mM Tris pH = 7.8 and 1 mM PMSF. Cells were left for 1 hour at room temperature for lyses, and then sonicated for 5 min. Total proteins from each extract were subjected to SDS-PAGE on a 10% or 12% acrylamide gel and transferred to a PVDF membrane. Western blot analyses were performed with a rabbit antiserum directed against GFP (Abcam) at a dilution of 1:5000, followed by a second Goat anti-rabbit HRP conjugate (Pierce) at a dilution of 1:5000. Proteins were visualized using an ECL-Plus kit (Amersham).

## Supporting Information

Table S1 Silicic acid responding genes in P. tricornutum.
(0.03 MB XLS)

Table S2 Mapping of InterPro domains of the differentially express genes.
(0.05 MB XLS)

Figure S1 Genetic organization of the PtSIT2 gene cluster. The full-length open reading frame of PtSIT2 genes is conserved (no mismatch at the nucleotide level) as well as 441 nt and 181 nt, at the 5′- and 3′-UTR regions (light grey), respectively. To check for the coherence of PtSIT2 duplication on the chromosome 18, we used a number of primers which allowed to obtain DNA fragments in the range 0.4-3.5 kb. The primers used are: #1 (5′-GTCAGTCAGAGAGAGTCACAC-3′), #3 (5′-ATGGCGGACGTTGCCAACATT-3′), #4 (5′-CATACACAGTAAAACATCCCC-3′), #5 (5′-CATACACAGTAAAACATCCCC-3′), #6 (5′-CCTGCAGAAGACGTACACA-3′), #7 (5′-CGCTTTGTAACTCGGAGGAG-3′), #8 (5′-GACTGAGATAACAGCTTGACG-3′), #9 (5′-GTCTTACGGTATTTCAGTCCG-3′), and #10 (5′-AACACAGAGCAGCTACATTTGG-3′). Note the presence of a putative retrotansposon (i.e., gag-pol-env) of the Ty1/Copia-like family. The oligonucleotides (half arrow) used to test for the PtSIT2 duplication are indicated.
(2.72 MB TIF)
