# Supplementary figures and images for: Genome-Wide Transcriptome Analyses of Silicon Metabolism in Phaeodactylum tricornutum Reveal the Multilevel Regulation of Silicic Acid Transporters
Source: PLoS One. 2009 Oct 14;4(10):e7458. doi: 10.1371/journal.pone.0007458 (PMC2758714; doi:10.1371/journal.pone.0007458)

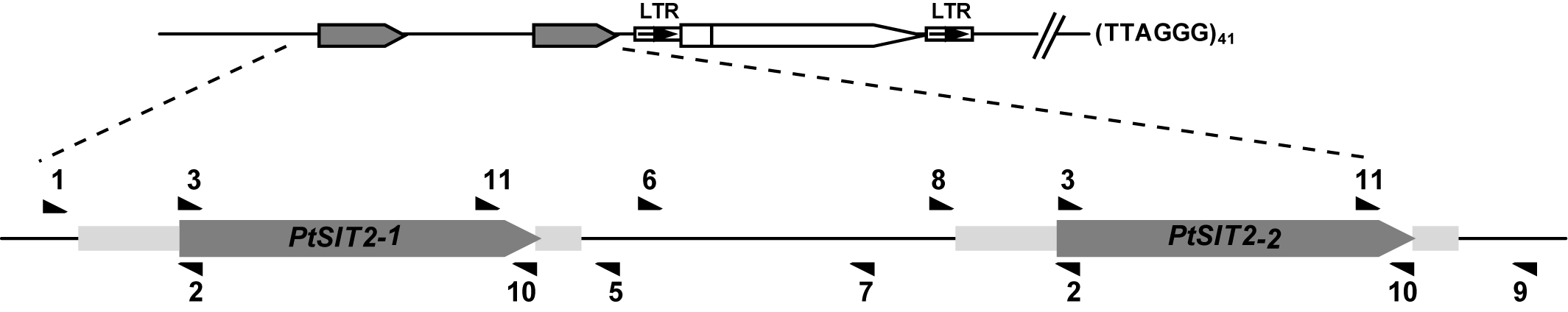

Supplement: Figure S1 — Genetic organization of the PtSIT2 gene cluster. The full-length open reading frame of PtSIT2 genes is conserved (no mismatch at the nucleotide level) as well as 441 nt and 181 nt, at the 5′- and 3′-UTR regions (light grey), respectively. To check for the coherence of PtSIT2 duplication on the chromosome 18, we used a number of primers which allowed to obtain DNA fragments in the range 0.4-3.5 kb. The primers used are: #1 (5′-GTCAGTCAGAGAGAGTCACAC-3′), #3 (5′-ATGGCGGACGTTGCCAACATT-3′), #4 (5′-CATACACAGTAAAACATCCCC-3′), #5 (5′-CATACACAGTAAAACATCCCC-3′), #6 (5′-CCTGCAGAAGACGTACACA-3′), #7 (5′-CGCTTTGTAACTCGGAGGAG-3′), #8 (5′-GACTGAGATAACAGCTTGACG-3′), #9 (5′-GTCTTACGGTATTTCAGTCCG-3′), and #10 (5′-AACACAGAGCAGCTACATTTGG-3′). Note the presence of a putative retrotansposon (i.e., gag-pol-env) of the Ty1/Copia-like family. The oligonucleotides (half arrow) used to test for the PtSIT2 duplication are indicated. (2.72 MB TIF) [file pone.0007458.s003.tif]
